# Sunlit zebra stripes may confuse the thermal perception of blood vessels causing the visual unattractiveness of zebras to horseflies

**DOI:** 10.1038/s41598-022-14619-7

**Published:** 2022-08-04

**Authors:** Péter Takács, Dénes Száz, Miklós Vincze, Judit Slíz-Balogh, Gábor Horváth

**Affiliations:** 1 Department of Biological Physics, ELTE Eötvös Loránd University, Pázmány sétány 1, Budapest, 1117 Hungary; 2 MTA-ELTE Theoretical Physics Research Group, ELTE Eötvös Loránd University, Pázmány sétány 1, Budapest, 1117 Hungary

**Keywords:** Biophysics, Physiology, Zoology

## Abstract

Multiple hypotheses have been proposed for possible functions of zebra stripes. The most thoroughly experimentally supported advantage of zebra stripes is their visual unattractiveness to horseflies (tabanids) and tsetse flies. We propose here a plausible hypothesis why biting horseflies avoid host animals with striped pelages: in sunshine the temperature gradients of the skin above the slightly warmer blood vessels are difficult to distinguish from the temperature gradients induced by the hairs at the borderlines of warmer black and cooler white stripes. To test this hypothesis, we performed a field experiment with tabanids walking on a host-imitating grey test target with vessel-mimicking thin black stripes which were slightly warmer than their grey surroundings in sunshine, while under shady conditions both areas had practically the same temperature as demonstrated by thermography. We found that horseflies spend more time walking on thin black stripes than surrounding grey areas as expected by chance, but only when the substrate is sunlit. This is because the black stripes are warmer than the surrounding grey areas in the sun, but not in the shade. This is consistent with the flies’ well-documented attraction to warmer temperatures and provides indirect support for the proposed hypothesis. The frequent false vessel locations at the numerous black–white borderlines, the subsequent painful bitings with unsuccessful blood-sucking attempts and the host’s fly-repellent reactions enhance considerably the chance that horseflies cannot evade host responses and are swatted by them. To eliminate this risk, a good evolutionary strategy was the avoidance of striped (and spotted) host animals.

## Introduction

Since the debate among Galton^1^, Wallace^2,3^ and Darwin^4^ between the 1850’s and 1870’s, multiple ideas have been proposed for possible functions of zebra stripes. The main hypotheses are the following^5,6^: (1) Apparent size increase^6–9^: The stripes could induce a visual illusion increasing the apparent size of zebras and could ensure an advantage over their predators. (2) Decreased visibility in twilight^1,7,8,10–12^: At dusk and dawn or in moonshine, stripes might be difficult to discern. (3) Dazzling the eyes of predators by moving stripes^12–16^: The moving stripes of escaping zebras may dazzle and confuse their predators, which could difficultly select an individual zebra from the herd. (4) Optical camouflage from predators^2–4,17–21^: Stripes may dissolve the animal contour and thus could blend in zebras with their background. (5) Intraspecific communication by individual stripe patterns^8,12,22–24^: Zebras could recognize each other by means of their individual stripe patterns, which can be visual markers for group bonding and/or particular body parts for grooming. (6) Warning colouration, or aposematism^6,17^: The conspicuous black-white striped pattern of zebras may offer protection by advertising antipredator defenses, such as dangerous kicks and bites, and thereby warn predators to avoid them. (7) Visual defence against blood-sucking tsetse flies and tabanids carrying pathogens of serious diseases^25–36^: Since tsetse flies and tabanids avoid striped targets and prefer homogeneous objects, they do not bite zebras as frequently as solidly coloured host animals. (8) Visual signalling of fitness^37^: Irregularities in the stripe pattern because of injuries or other dysfunctions could visually signal the poor physical condition of a zebra for mate-seeking conspecifics. (9) Cooling by convective air eddies above sunlit stripes^8,12,22,38,39^: Sunlit black and white stripes may induce rotary convections, which air eddies could cool the striped animal.

In field experiments Blahó et al.^33,40^, Egri et al.^32^ and Horváth et al.^41^ have shown the following: (i) Female horseflies rarely attack host animals with a coat of inhomogeneous (striped or spotted) intensity and/or reflection-polarization patterns. The attractiveness to tabanids quickly decreases with increasing inhomogeneity (increasing number and decreasing width of stripes/spots) of the coat, and below a critical stripe/spot width (= threshold) the target becomes unattractive to horseflies relative to a homogeneous coat. The stripe widths of the three living zebra species (*Equus zebra*, *E. burchelli*, *E. grevyi*) fall below this threshold. The exact reason for the tabanid-repellency of inhomogeneous coat patterns is unknown^21,34^. In this work, we propose a new explanation, which does not exclude the validity of the earlier ones. (ii) Zebra-striped patterns do not lose their tabanid-unattractiveness even if tabanid-attractive gases (CO_2_, ammonia) are emitted to an extent that is characteristic to the host animals. Thus, the visual tabanid-repellency of zebra stripes overwhelms the attractiveness of zebra-specific odours. (iii) In the case of sunlit targets with inhomogeneous patterns, the few attracted female horseflies land mainly on dark stripes/spots instead of bright ones. The main reason for this behaviour is that in sunshine dark surfaces are warmer than bright ones, and tabanids prefer warmer host animals^42^, because they can escape from warmer targets more easily to avoid the parasite-repellent reactions of the attacked host^43^.

In a thermodynamic field experiment, Horváth et al.^44^ found no evidence for cooling by stripes, and using schlieren imaging (i.e. visualization of spatio-temporal changes of the local refractive index of transparent media by grey shades/streaks^45^) in laboratory experiments, Pereszlényi et al.^46^ demonstrated that cooling air eddies do not form above sunlit zebra stripes. Both findings contradict theory 9 mentioned above. According to Caro et al.^47^, Caro and Stankowich^48^ and Caro^6^, the most important selection factor in the evolution of zebra stripes is the defence against tabanids, which results support theory 7. Kojima et al.^49^ showed that black cows painted with white stripes imitating zebra stripes minimally attract blood-sucking stable flies (*Stomoxys calcitrans*), horn flies (*Haematobia irritans*) and tabanids (*Tabanus sapporoensis*). This observation also supports theory 7. Currently, the most thoroughly proven evolutionary advantage of zebra stripes and other striped/spotted coat patterns is the visual unattractiveness to biting flies, especially horseflies and tsetse flies^6,26,27,31–33,40,41,49^. However, it is still unclear why striped/spotted host animals are unattractive to female blood-seeking tabanids.

Melin et al.^21^ suggested that compared to the homogeneous pelage of other sympatric herbivores, it seems highly unlikely that zebra stripes serve as antipredator camouflage. Caro et al.^34^ showed that up close, striped surfaces prevent horseflies from making a controlled landing. As a consequence, very few tabanids land on zebras, stay a long time, or probe for blood. How et al.^36^ found that horseflies avoid landing on, fly faster near, and do not approach as close to striped and checked targets compared to homogeneous grey ones, as has already demonstrated Waage^27^ experimentally.

In this work, we propose the following new explanation for this phenomenon: A blood-seeking female horsefly landed on a host animal should find a blood vessel directly below the host's body surface. This may be performed by her thermoreceptors^50^ on the legs^51^ or antennae^52^ or mouthpart^53^ which sense the slightly higher skin temperature above a vessel relative to its surroundings. Thus, vessel location could happen with thermoreception by means of vessel-induced temperature gradients. Tabanids evidently prefer sunlit, warm host animals and targets^32,33,40,42,54–56^. Horváth et al.^43^ showed that biting horseflies prefer a warmer host, because they can escape easier from a warmer target compared to a colder one. If the sunlit coat of a host is striped, on this inhomogeneous pattern all borderlines of adjacent dark and bright stripes have temperature gradients (differences), because in sunshine the dark stripes are warmer than the bright ones^6,39,44,57–59^. The temperature gradients at the borderlines deceptively may imitate vessel-induced temperature differences for a blood-seeking female horsefly, if she looks for vessels by thermoreception. Thus, the borderlines of sunlit black and white stripes may hamper successful thermal vessel detection. According to our hypothesis, tabanids do not prefer to land on striped (or spotted) hosts, because on such targets it is difficult to locate vessels by thermoreception due to the disruptive temperature gradients of the numerous borderlines of black and white stripes in sunshine. Therefore, female horseflies wanting to suck blood attack less frequently striped host animals, because on homogeneous body surfaces, the temperature gradients above blood vessels can be detected more easily than on sunlit striped patterns.

To test this hypothesis, we performed a field experiment with tabanids: We attracted female horseflies by a medium grey barrel with thin black stripes mimicking a visually attractive host. The thin horizontal and vertical black stripes on the barrel’s cylinder imitated thermally the slightly warmer subsurface blood vessels when the studied half of the cylinder was sunlit. When the striped cylinder’s half was shady, the temperature of the thin black stripes was practically the same as that of their grey surroundings. We measured the total time periods of alighted and walking horseflies spent on the grey and the black areas of the sunlit or shady cylinder. Our aim was to elicit whether tabanids prefer the black stripes only if they are sunlit and thus warmer than their grey surroundings. If the stripes were preferred to the grey areas only under sunlit conditions, this preference could be explained exclusively with their higher temperature, rather than with their darkness and/or higher polarization. This would corroborate the supposed thermal vessel recognition in horseflies, and could support that sunlit stripes hamper the thermodetection of host’s vessels by tabanids.

## Materials and methods

### Ethics declarations

Our field experiment was performed with horseflies (Tabanidae), which blood-sucking parasites are non-protected insects in Hungary. Furthermore, as a demonstration, we recorded the thermograms of a horse with the permission of the horse owner (Mr. István Simon). For our purpose (i.e. to show the fine temperature gradients of the hairy skin above blood vessels), one such demonstration on a single horse was enough. This thermography was passive, that is the infrared radiation originating from the hairy horse skin was recorded by a thermal camera from a distance without any contact of the horse. From the viewpoint of the horse, this undangerous thermal recording was equivalent with a common photography, for which no approval from an ethical committee is necessary. We confirm the followings: (i) For both studies no institutional permission, licence or approval were necessary. (ii) No animals (neither horseflies, nor horses) were killed specifically for the purpose of these studies.

### Field experiment with a black-striped grey barrel

**Figure 1:**
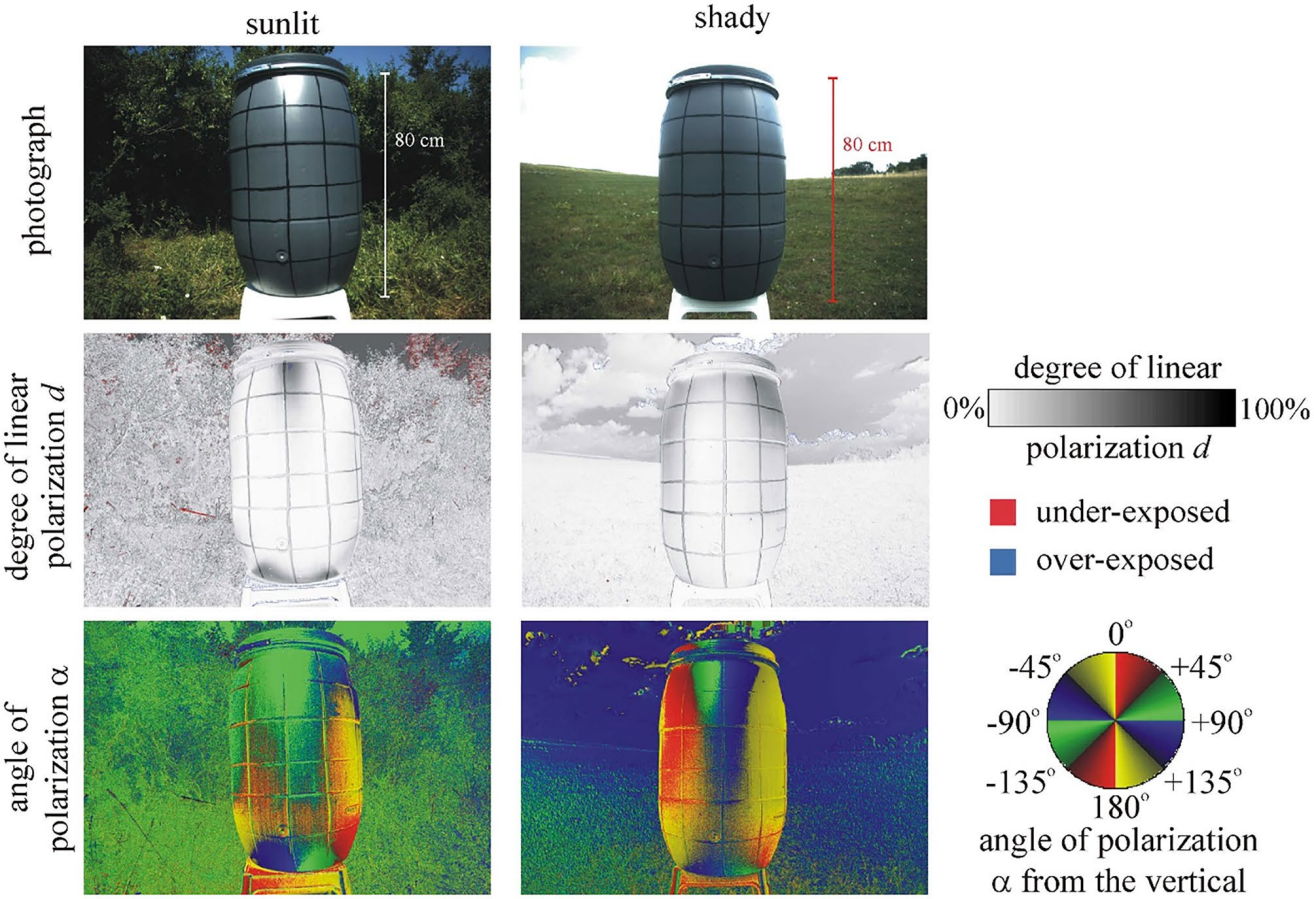
Reflection-polarization patterns of the sunlit (left column) and shady (right column) black-striped grey barrel used in the field experiment measured with imaging polarimetry in the green (550 ± 40 nm) spectral range when the optical axis of the polarimeter was horizontal. More details of the reflection-polarization characteristics of this target can be seen in Supplementary Figs. S2 and S3.

**Figure 2:**
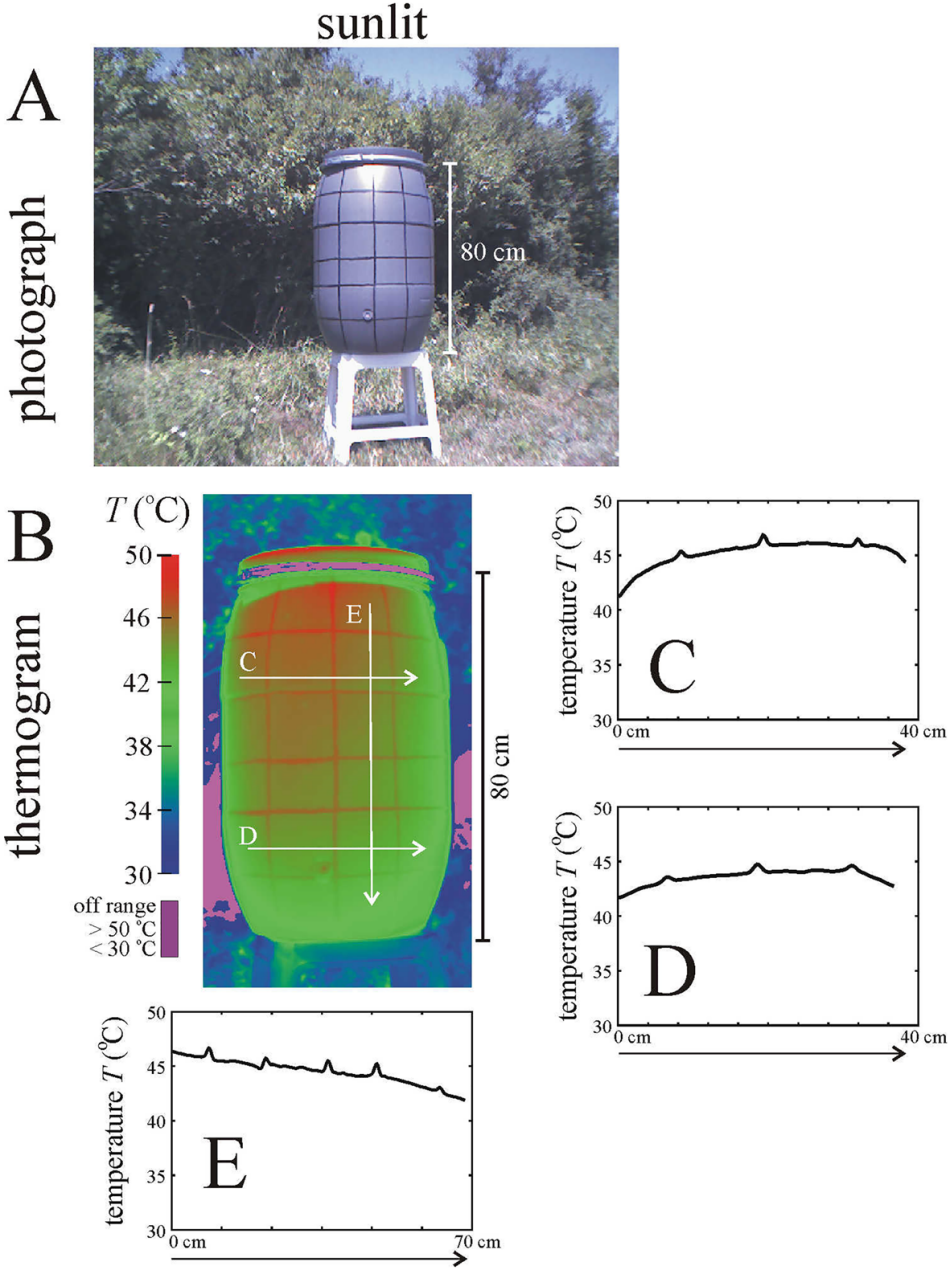
Photograph (**A**) and thermogram (**B**) of the sunlit black-striped grey barrel used in the field experiment. The barrel’s surface temperature *T* (°C) along the horizontal and vertical white arrows in the thermogram are shown in panels (**C–E**).

**Figure 3:**
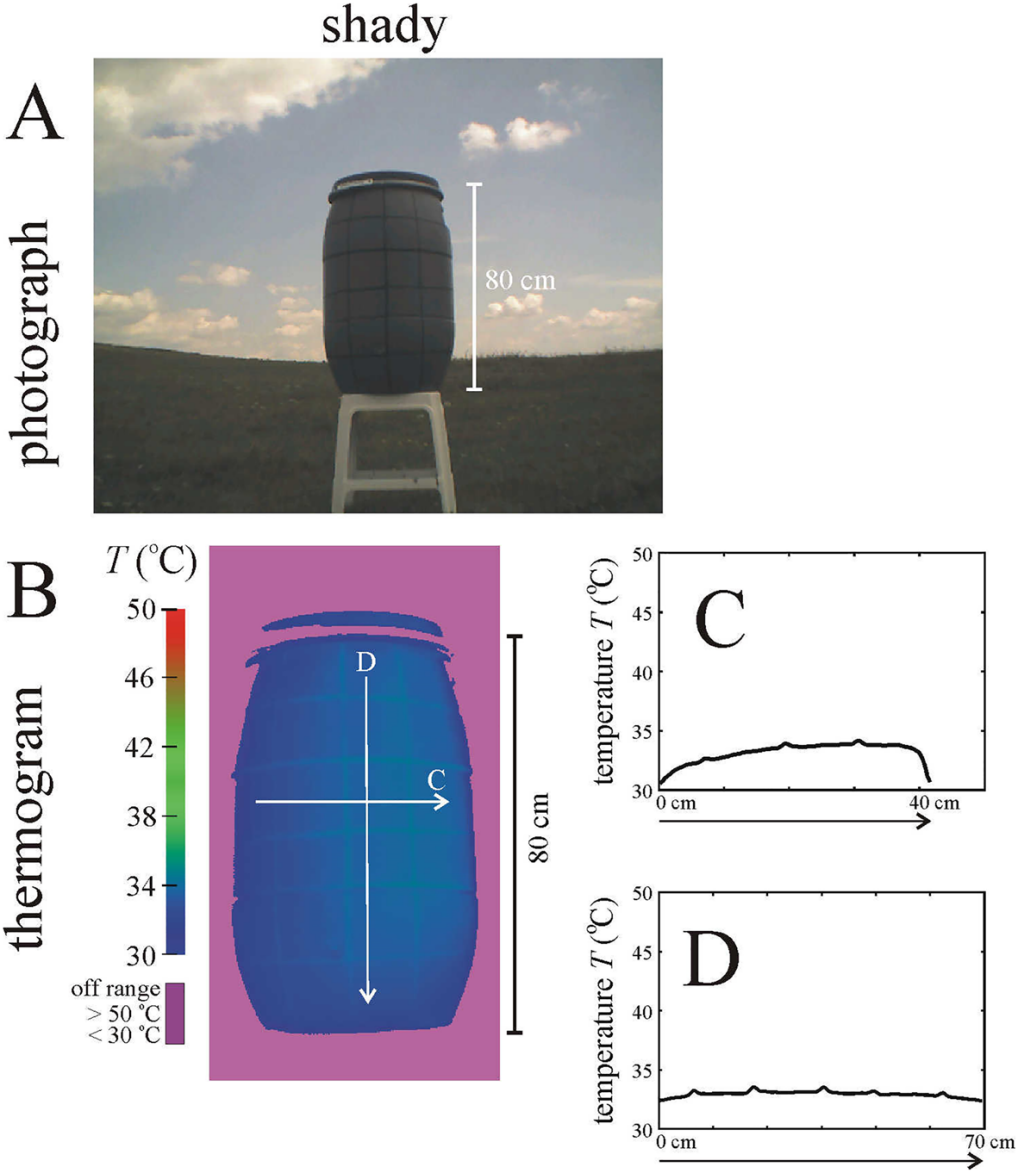
Photograph (**A**) and thermogram (**B**) of the shady black-striped grey barrel used in the experiment. The barrel’s surface temperature *T* (°C) along the horizontal and vertical white arrows in the thermogram are shown in panels (**C,D**).

Our experiment was performed from 7 July to 9 August 2021 on a horse farm in Szokolya (47° 52' North, 19° 00' East, Hungary) with an abundant horsefly population, where several other experiments with tabanids have been conducted in the last decade^32,42,54,60–62^. On the experimental days (Supplementary Table S1) the weather was sunny and warm (25–38 °C) with some cumulus clouds in the afternoon. The aim of this experiment was to imitate the slightly higher temperature of the skin over blood vessels of host animals (e.g. zebras and other ungulates) of tabanids. The dark host’s body was modelled by a medium grey (greyness *g* = 50%) cylindrical plastic (with low thermal conduction to keep the gradients of surface temperature) barrel (height *h* = 80 cm, radius *r* = 22 cm), and the vessels were imitated with 10 vertical and 5 horizontal black, thin (width *w* = 0.5 cm) stripes 13 cm apart (see the photograph of Figs. 1, 2, 3) composed of a common oil-based black paint. In sunshine these stripes were slightly warmer than their grey surroundings (which was shown by thermography), because the formers had greater light absorbance (smaller albedo) than the latters. Note that tabanids can sense the temperature of the grey or black regions of the barrel’s surface only when they contact them after landing.

**Figure 4:**
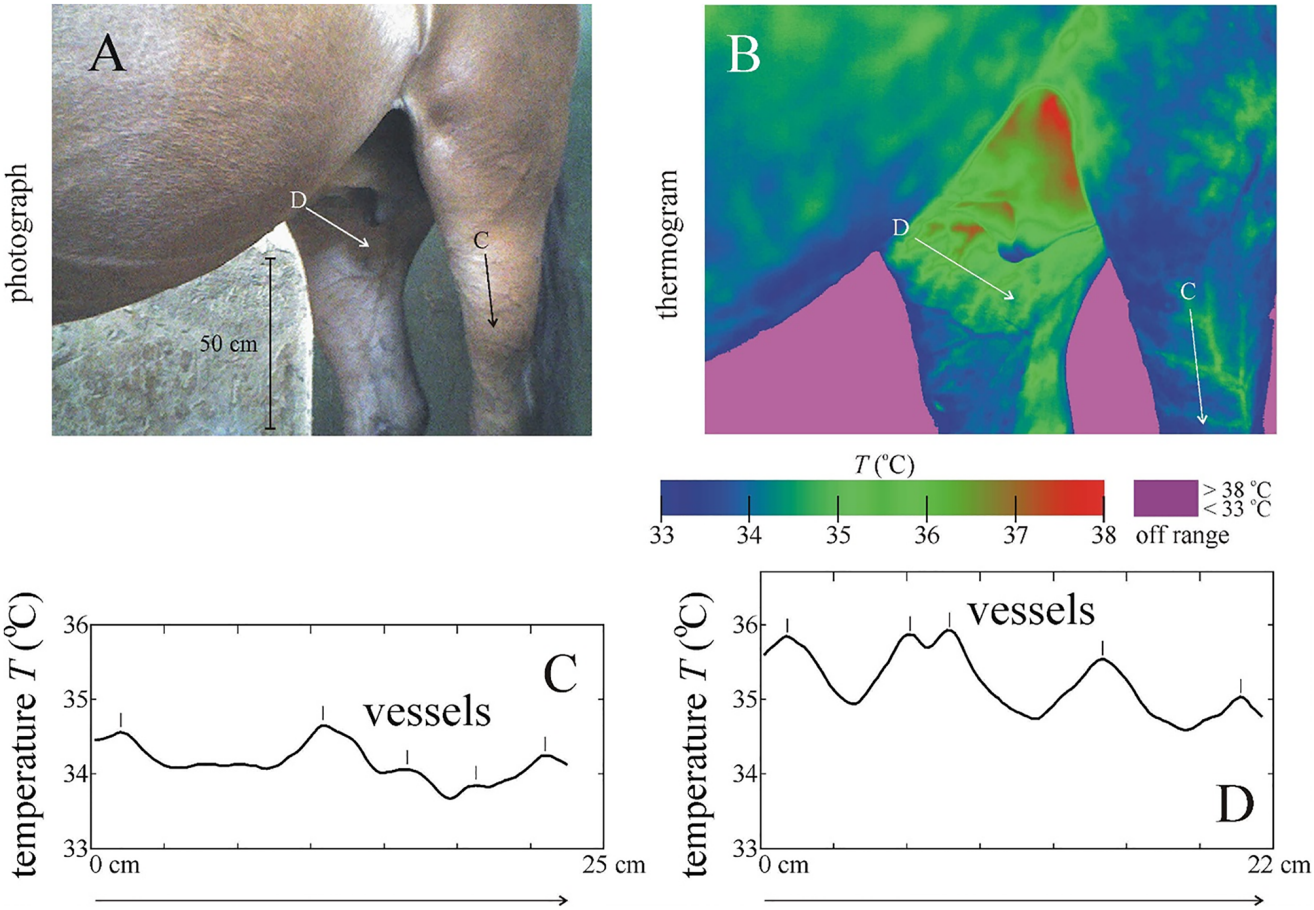
Photograph (**A**) and thermogram (**B**) of a shady brown horse at ambient air temperature of 35 °C. (**C,D**) Surface temperature *T* (^o^C) along arrows (**C,D**) in the photo and thermogram. In panels (**C–E**) the vessels are marked by vertical bars.

**Figure 5:**
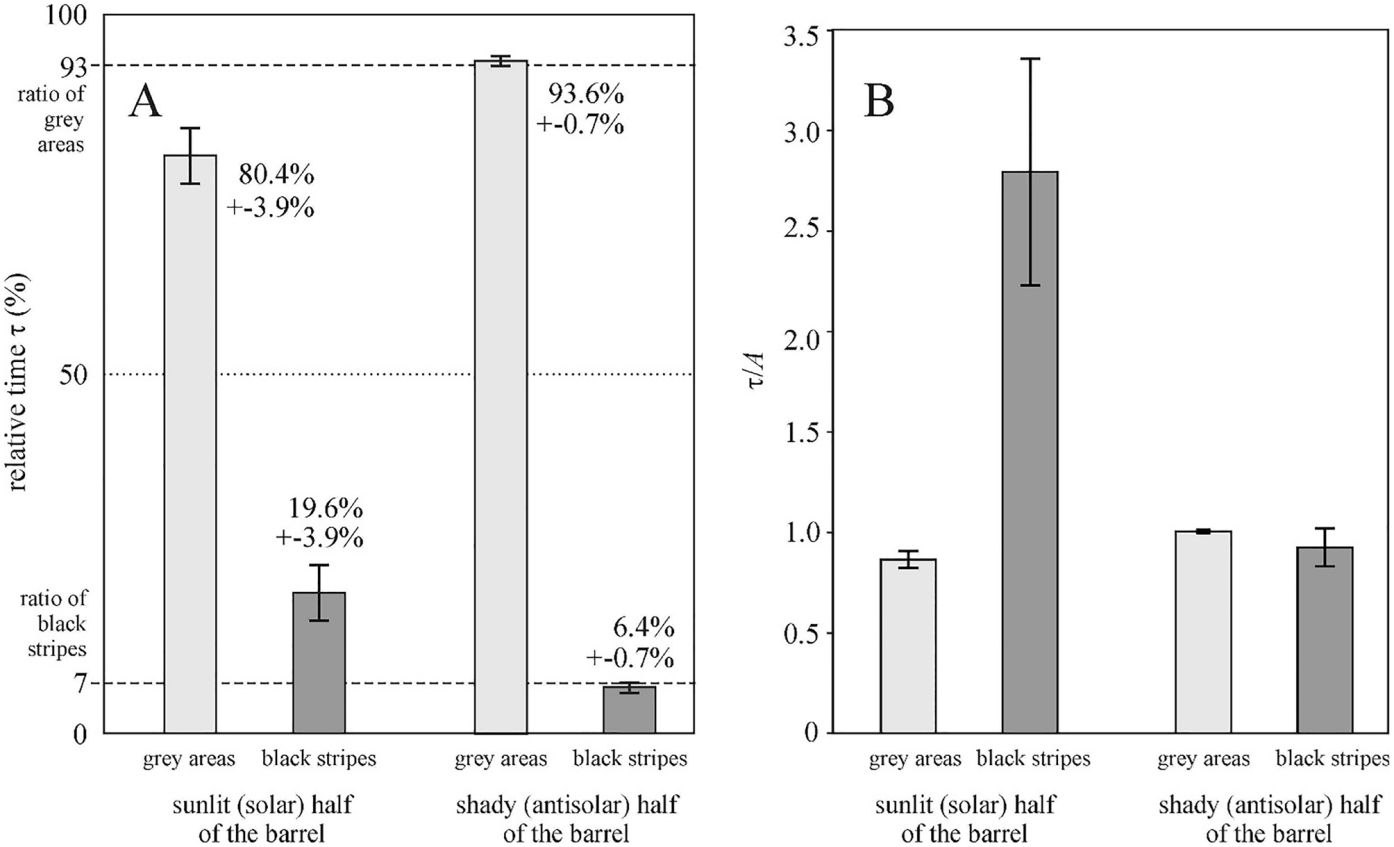
(**A**) Mean ± standard deviation (%) of the relative times τ_grey_ = *t*_grey_/(*t*_grey_ + *t*_black_) and τ_black_ = *t*_black_/(*t*_grey_ + *t*_black_) spent by female tabanids walking on the grey areas (*t*_grey_) and the black stripes (*t*_black_) of the sunlit (solar) and shady (antisolar) halves of the barrel’s cylinder in the field experiment (Supplementary Table S1). Relative area *A*_grey_ = 93% of the half cylinder was grey, and relative area *A*_black_ = 7% was covered with thin black stripes. (**B**) Mean ± standard deviation of the relative-area-normalized relative times τ_grey_/*A*_grey_ and τ_black_/*A*_black_ (for statistics see Table 1).

Although our test target (black-striped grey barrel) is very artificial relative to the real host animals of horseflies, we did not measure the spectral reflectance of the black stripes of our grey barrel, and did not compare it with that of black stripes of zebras measured earlier in another field experiment (see Supplementary Fig. S10 of^44^). Such a comparison was unnecessary, because in our present field experiment, the most important aspect was to imitate the fine temperature gradients of the host’s skin above blood vessels and at the borderlines of sunlit adjacent black and white zebra stripes, by adding sunlit thin black stripes to the grey barrel. According to the thermograms presented in this work (Figs. 2, 3, and 4, Supplementary Fig. S1) as well as in Figs. 3 and 5 of^44^, this imitation was appropriate.

The surface area of the half barrel’s cylinder (which was monitored by a person, see later) is *A*_m_ = *r*π*h*, and on it there were five vertical black stripes (Fig. 1) each with length *h* and width *w*. The surface area of the five vertical stripes is *A*_v_ = 5*hw*. In the half cylinder ran also five horizontal black stripes (Fig. 1) each with length *r*π and width *w*. The surface of the 5 horizontal stripes is *A*_h_ = 5*r*π*w*. The portion of the total area of the black stripes relative to the area of the half cylinder is *Q*_bs_ = (*A*_v_ + *A*_h_)/*A*_m_ = 5*w*(*h* + *r*π)/(*r*π*h*) ≈ 0.07, where *w* = 0.5 cm, *h* = 80 cm and *r* = 22 cm. The portion of the total area of the grey rectangles between the black stripes relative to the half cylinder is *Q*_gr_ = (*A*_m_ − *A*_v_ − *A*_h_)/*A*_m_ = (*r*π*h* − 5*hw* − 5*r*π*w*)/(*r*π*h*) ≈ 0.93. Hence, 93% of the half cylinder was grey, and only 7% was covered with the vessel-imitating thin black stripes.

The black-striped grey barrel was placed on a white tetrapodal plastic stool (height = 46 cm), and the tabanids landing and walking on the barrel were observed by one (always the same) person from a distance of 2 m (Fig. 1). A landed tabanid (which was always female, see “Results”) either stayed in one place for a period before she flew away, or walked around with some stops before flying away. During walking she frequently swept the substrate with her two forelegs. This typical periodic sweeping-touching motion of the forelegs might have served to test the quality (e.g. temperature, odour, flavour, stiffness/softness, roughness/smoothness, wetness) of the substrate, and also to detect the surfacial temperature gradients over blood vessels of the host’s skin.

The observer was sitting on a chair and wore white clothes and a bright hat against sunshine and to reduce his visual attractiveness to tabanids. He observed either the sunlit (solar: facing toward the sun) or the shady (antisolar: facing toward the antisolar point) half of the barrel’s cylinder. His direction of observation followed (changing in every 30 min) the solar-antisolar meridian. He was interested only in the walking tabanids which obviously monitored the substrate and looked for something. Using two stop-watches, the observer measured the time periods spent by the walking tabanids on the grey rectangles (with a stop-watch held in the right hand) and the black stripes (with a stop-watch held in the left hand) of the barrel’s surface. When a female tabanid landed on the grey/black area and started to walk, the right/left stop-watch was started and was stopped when she flew away, or walked onto a black/grey area. This procedure was repeated throughout the whole session of observation on a given day from morning to afternoon. If after alighting a tabanid stayed in one place before flying away, her staying period was not registered with the stop-watch, because this reaction was irrelevant with respect to vessel seeking.

Since we aimed to measure the time periods spent by tabanids walking on the grey and black regions of the barrel’s surface, a non-sticky (dry) barrel was used. Thus, the same tabanid individual might have landed more than once on the barrel. Such pseudoreplication could have been avoided only with a sticky barrel, on which an alighted tabanid could not have walked and flew away, consequently the time period spent by the fly on the barrel could not have been measured.

### Imaging polarimetry

Since horseflies are polarization sensitive^63,64^ and female tabanids are polarotactic insects (i.e. they are attracted to linearly polarized light during host seeking^65^) we measured the reflection-polarization characteristics of the test black-striped grey barrel used in our field experiment with imaging polarimetry in the red (650 ± 40 nm), green (550 ± 40 nm) and blue (450 ± 40 nm) parts of the spectrum^66,67^. These polarization patterns helped to interpret the tabanid attractiveness of this target.

### Thermography

The temperature distributions (thermograms) of the half cylinder of the black-striped grey barrel used in the experiment were measured with a thermal camera (VarioCAM®, Jenoptik Laser Optik Systeme GmbH, Jena, Germany) having a nominal precision of ± 1.5 °C, which was validated by calibration with a contact thermometer (GAO Digital Multitester EM392B 06554H, EverFlourish Europe Gmbh., Friedrichsthal, Germany) possessing a nominal precision of ± 1 °C (see the Electronic Supplementary Materials of^42^).

The horizontal optical axis of the thermal camera was perpendicular to the vertical barrel, and the air temperature was about 35 °C. The thermograms of the sunlit (solar) or shady (antisolar) half of the barrel’s cylinder were registered under a clear sky in sunshine. These thermograms served as a demonstration of the typical temperature patterns of our test targets under sunny conditions when host-seeking female horseflies are very active. There was no need to capture the thermograms continuously throughout each experimental day, because this experiment was performed always under a cloudless sky in full sunshine, when the thermograms were qualitatively very similar: on the sunlit half of the barrel’s cylinder the black stripes were always slightly warmer than the surrounding grey areas, while on the shady half the black stripes had practically the same temperature as their grey surroundings (see later).

We also recorded thermograms of the hairy body surface of a brown horse in a horse farm (Göd, Hungary) with the permission of the farm owner (Mr. István Simon). Since this thermographic recording was quite undangerous for the horse, we did not need permission or approval from an institutional or licensing committee. We chose a brown horse, rather than a white or a black one, because among the natural hosts (mainly ungulates) of horseflies in Africa, where zebras live, there are neither white, nor black animals. White (albino) animals are quickly selected out due to their striking bright appearance under any illumination conditions, while black animals could not tolerate the heat in sunshine. Natural hosts typically have medium bright/dark grey or brown fur. Earlier, we have measured the thermograms of sunlit and shady living zebras in a zoo^44^ and zebra hides in a laboratory^46^. Furthermore, for the thermographic demonstration of the temperature gradients of the shady horse skin above the slightly warmer blood vessels, a horse of any colour is appropriate.

The surface temperature *T* along straight lines on the target surfaces was determined with our custom-developed software. On the thermograms horizontal, or vertical, or tilted lines were selected, along which the average and standard deviation of *T* were calculated.

Our experiment was conducted in Hungary (at a latitude of 47°52'N), while zebras live in Africa, around the Equator. The intensity of celestial radiation (sun- and skylight) is different between these two regions. In spite of this fact, we did not measure the intensity *I* of sunlight on the experimental days, because this was unnecessary. For our experiment the following criteria were the only important: (i) Flying blood-seeking female tabanids were abundant in the field. (ii) The test surface (half cylinder of the black-striped grey barrel) was either sunlit or shady. Thus, it offered similar gradients of the surface temperature *T* for landing female tabanids as *T*-gradients forming on the coat of sunlit or shady zebras. This was demonstrated by comparison of the thermograms of our test surface and those measured earlier on living zebras and zebra hides (see Figs. 3 and 5 of^44^, and Fig. 4 of^46^). (iii) These *T*-gradients were measured and visualized by our thermocamera. All these made superfluous the measurement of *I* of sun- and skylight illuminating our test target. The result, that is the *T*-gradient induced by the ambient radiation (sun- and skylight) was the relevant point, rather than the *I* of illumination causing it.

### Statistics

In the experiment, we compared the ratios of total time tabanids spent on the black stripes to the surface ratio of black stripes on the barrel. We calculated the mean and standard deviation of the measured time ratios. Then, we divided these time ratios with the corresponding (grey or black) area of the barrel to get relative time values, independent of the grey or black parts of the barrel. Using Student’s t-test for the sunlit and shady half of the barrel, we compared the relative times for the black and grey areas. We chose a significance level of p = 0.01. For statistical computations, we used the statistical function package of Microsoft Excel 2021.

## Results

### Time periods walking horseflies spent on the shady and sunlit halves of the black-striped grey barrel

Female horseflies have an ommatidia-free thin dorsal zone between their two compound eyes, while in males both eyes connect dorsally. These characteristics of the tabanid eyes could have been observed in our experiment, in which only female tabanids alighted on the black-striped grey barrel. According to Fig. 5A (Supplementary Table S1), the females walking on the shady antisolar half of the black-striped grey barrel spent τ_black_ = 6.4% and τ_grey_ = 93.6% of their total time on the black stripes and the grey areas, respectively. The average time ratio that female tabanids spent on the black stripes of the shady half of the barrel’s surface was 6.4 ± 0.7% (mean ± standard deviation). This ratio is approximately the same as the surface ratio *A*_black_ = 7% of the black stripes, since 7% is within the scattering range of the average time ratio. Since the surface percentage of the black and grey areas is *A*_black_ = 7% and *A*_grey_ = 93%, respectively, we conclude that the walking tabanids preferred neither the shady black stripes, nor the shady grey rectangles.

On the other hand, the average time ratio that walking female tabanids spent on the black stripes of the sunlit half of the barrel’s cylinder was τ_black_ = 19.6% ± 3.9% (Fig. 5A, Supplementary Table S1) which is 2.8 times more time than the surface ratio *A*_black_ = 7% of the black stripes. From this we conclude that walking horseflies preferred the sunlit black stripes to the sunlit grey rectangles.

This conclusion is corroborated by Fig. 5B, which shows the mean ± standard deviation of the relative-area-normalized relative times τ_grey_/*A*_grey_ and τ_black_/*A*_black_. According to Table 1, on the sunlit half of the barrel, τ_black_/*A*_black_ on the black stripes was significantly higher than τ_grey_/*A*_grey_ on the grey rectangles. However, on the barrel’s shady half, there was no statistical difference between τ_black_/*A*_black_ and τ_grey_/*A*_grey_ at significance level of p = 0.01.

**Table 1:** Statistical analysis between the relative-area-normalized relative times τ_grey_/*A*_grey_ and τ_black_/*A*_black_, where τ_grey_ = *t*_grey_/(*t*_grey_ + *t*_black_) and τ_black_ = *t*_black_/(*t*_grey_ + *t*_black_) are the relative times spent by female tabanids walking on the grey areas (*t*_grey_) and the black stripes (*t*_black_) of the sunlit (solar) and shady (antisolar) halves of the barrel’s cylinder in the field experiment, *A*_grey_ = 93% is the relative area of the half cylinder that was grey, and *A*_black_ = 7% is the relative area that was covered with thin black stripes.

	Sunlit half of the barrel's cylinder		Shady half of the barrel's cylinder	
	p-value (t-test)	Significance (p < 0.01)	p-value (t-test)	Significance (p < 0.01)
Black versus grey	0.0000	Significant	0.0339	Not significant

### Reflection-polarization characteristics of the black-striped grey barrel

According to Fig. 1 (Supplementary Figs. S2-S3), the degree of linear polarization *d* of light reflected from the black stripes of the grey barrel used in the field experiment was always higher (55% ≤ *d* ≤ 67%) than that reflected from the grey rectangles (*d* ≤ 24%) between the stripes under both sunlit and shady conditions. The reason for this is the law of Umow^68^: the darker a surface in a given spectral range, the higher is the degree of polarization of reflected light. Apart from two highly polarized patches (43% ≤ *d* ≤ 51%) at the uppermost and lowermost regions of the barrel’s cylinder (from which light was reflected nearly by the Brewster’s angle for which reflected light has maximal *d*), the sunlit grey rectangles reflected light with lower *d* (*d* ≤ 15%) than the shady ones (*d* ≤ 24%). The angle (direction) of polarization α of light reflected from a surface is always perpendicular to the plane of reflection determined by the dominating incident ray of sun- and/or skylight, the point observed and the observer’s viewing direction (optical axis of the polarimeter). Therefore, both the left and the right vertical sides of the barrel reflected nearly vertically polarized light, because the plane of reflection was nearly horizontal there. On the other hand, the central vertical zone of the barrel’s cylinder reflected mainly horizontally polarized light, since the plane of reflection was approximately vertical in that case. These reflection-polarization patterns of the black-striped grey barrel imitated well those of sunlit and shady zebra hides and zebra-striped test surfaces used in our earlier field experiments^32,33,35,40,41^.

Since host-seeking female tabanids are strongly attracted by high *d*-values, independently of α^41,60^, one could have expected that in our experiment the landing and walking tabanids always preferred visually the black stripes of the grey barrel. In the subsection “Time periods walking horseflies spent on the shady and sunlit halves of the black-striped grey barrel” we showed that there was no such a preference on the shady half of the barrel, while walking horseflies preferred the black stripes only on the barrel’s sunlit half. In our opinion, this finding is based on the temperature distribution of the (shady/sunlit) barrel’s cylinder (see later).

### Thermograms of the black-striped grey barrel and a brown horse

Under sunlit conditions, the black stripes of the grey barrel used in the field experiment were about 1.5–2 °C warmer than the grey areas because of the lower albedo of black areas relative to the higher albedo of the grey ones (Fig. 2, Supplementary Fig. S1). Under shady conditions, the black stripes were only negligibly warmer (by 0.15–0.2 °C) than the grey areas (Fig. 3, Supplementary Fig. S1). The temperature distribution of the black-striped grey barrel plays an important role in the interpretation of time periods that horseflies spent walking on the barrel’s cylinder (see later).

Figures 4A,B show the photograph and thermogram of a shady brown horse at 35 °C air temperature. The shadowed pelage regions above vessels were slightly warmer than the surrounding pleage areas, because the warmer vessels heated the colder pelage (Fig. 4C,D). Note, however, that these characteristics demonstrate the thermal distribution of the outermost pelage surface, rather than that of the skin surface below the hairs.

## Discussion

With our experimental setup, we showed that blood-seeking female horseflies landing on an artificial test surface mimicking a visually attracting host animal prefer fine gradients of surface temperature *T* (with tiny local *T-*differences restricted to thin stripes) imitating thermally either blood vessels or borderlines of sunlit neighbouring dark and bright stripes. This finding proves that in sunshine the *T-*gradients induced by the hairs at the borderlines of warmer black and cooler white zebra stripes can be confused with the *T-*gradients of the skin above the slightly warmer blood vessels. All these experimentally corroborate our new hypothesis that biting horseflies avoid host animals with striped pelages (e.g. zebras) because of the risk of such thermal confusions, which reduce the chance of a successful vessel finding.

From a remote distance, horseflies are attracted to their host animals by visual and olfactorial cues^29,53,55,69–76^. After landing on a host, their behaviour is also influenced by the temperature distribution of the host’s body surface^42,43,53,77,78^. In this work, we proposed a new explanation of the unattractiveness of zebra stripes to blood-seeking horseflies and presented the results of a field experiment which support our proposal.

### Thermal characteristics of a hairy horse skin

Currently, thermography is routinely used in equine sport and veterinary medicine as an efficient diagnostic tool for detection and monitoring of injuries, diseases, inflammations and other pathological areas of the musculoskeletal system and the hairy skin of horses^79^. Both internal and external factors have a significant influence on the body surface temperature *T* of horses. The *T*-pattern depends on the movement of the animal, as the skin overlying muscle has an increased *T* due to muscular activity. Because of evaporative cooling, sweating also affects *T*. At low ambient temperatures the subcutaneous vasculature constricts resulting in the cooling of the skin surface, while at high ambient temperatures it dilates resulting in the warming of the skin surface^79^.

The hair layer is a good thermal insulator blocking heat emission from the underlying skin. The equilibrium temperature of the hairy skin surface is determined by the balance between the heat loss by radiation and convection to the air, and the heat conduction through hairs^57^. In direct sunlight, black-hair-covered skin areas are much warmer than white(unpigmented)-hair-covered ones, while in shade there is no significant *T*-difference between black and white hairy areas^44,46^. The *T* of the hairy body surface exposed to sunlight depends on the shape, posture, curvature of the surface as well as on the variations in hair thickness and colour (pigmentation)^57^.

For the interpretation of the results presented in this work, the most important thermal characteristics of the hairy skin surface of horses are the following^43,79,80^: The thermograms of horses are related to the vasculature and tissue metabolism and is influenced by the ambient air temperature. The skin overlying major blood vessels is always warmer than skin areas farther from these vessels. Normally, the skin surface above veins is warmer than above arteries, because the former are in metabolically active areas and closer to the skin surface. Venous drainage from tissue with a high metabolic rate is warmer than venous drainage from normal tissue. Areas of increased skin temperature follow the vascular pattern, while areas distant from vessels are cooler.

In earlier field studies Horváth et al.^42,43^ recorded several thermograms of black, brown, beige and white horses with a thermal camera under sunny and shady conditions. These thermograms and Fig. 4 corroborate some of the above-mentioned thermal characteristics of the pelage of horses. In our present field experiment, horseflies were attracted to the warmer sunlit black stripes of the grey barrel mimicking the always warmer skin surface above vessels, independently of whether the hairs are sunlit or shady.

### Right or false thermal vessel detection on skins with homogeneous or striped pelage

The temperature distribution of the skin surface covered by hairs can be sensed by vessel-seeking female horseflies landed and walking on horses only if their thermoreceptors contact directly the skin. Therefore, the flies have to penetrate between the hairs to reach the skin surface.

**Figure 6:**
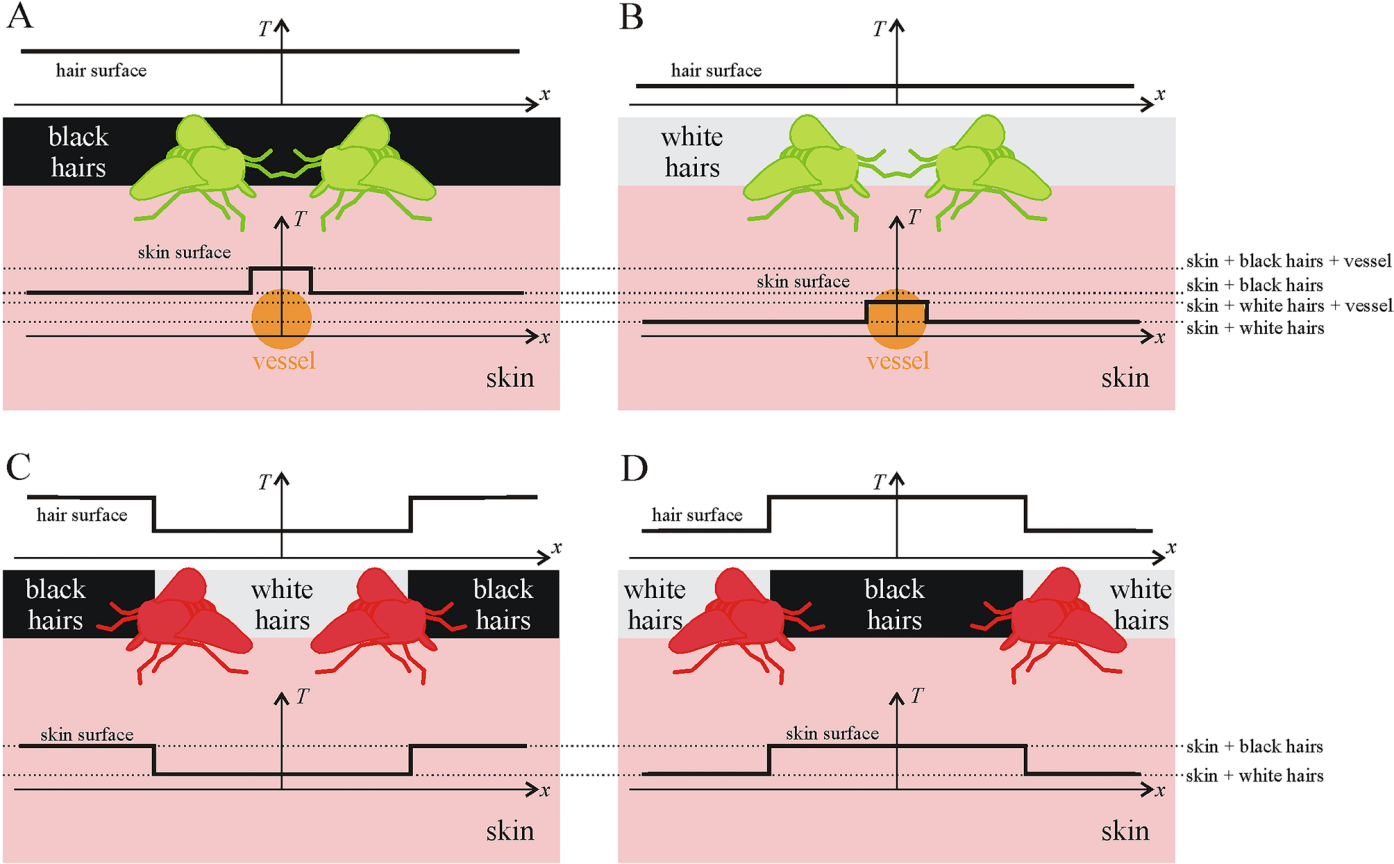
Situations of thermal detection of blood vessels (orange circles) by blood-seeking female horseflies on the sunlit hairy skin of host animals with homogeneous (**A,B**) and striped (**C,D**) pelage patterns. (**A**) Homogeneous black pelage with a vessel next to the skin surface. (**B**) Homogeneous white pelage with a vessel next to the skin surface. (**C,D**) Black and white striped pelage without a vessel in the skin. The inset graphs show qualitatively the spatial change of temperature *T* of hairs and skin surface along a line *x*, which crosses the borderlines of neighbouring black and white stripes in panels (**C,D**). The horizontal dotted lines show the four typical *T*-levels of the skin surface at skin (i) with white hairs < (ii) with white hairs above a vessel < (iii) with black hairs < (iv) with black hairs above a vessel. Green horseflies represent the sites of successful blood-sucking attempts induced by the positive *T*-gradients (increasing *T*) of the skin surface above vessels, while red horseflies represent the sites of unsuccessful blood-sucking attempts induced by the positive *T*-gradients of the skin surface below the borderlines of black and white hair stripes. The head of horseflies turns toward positive *T*-gradients. The dimensions of horseflies, vessels and thickness of the hair layer are not necessarily to scale.

Figure 6 demonstrates the relevant situations of thermal vessel detection by blood-seeking female horseflies on the sunlit hairy skin of host animals with homogeneous or striped pelage patterns. In sunshine, the temperature *T* of black hairs is always larger than *T* of white hairs. Due to the heat conduction of the hair layer, the hair-specific *T*-characteristics are transferred down to the skin surface, which is heated better by the warmer black hairs than by the cooler white hairs. On the other hand, independently of hairs, the skin surface above a blood vessel is slightly warmer than the surrounding areas, because the blood is slightly warmer than the surrounding tissues. The interaction of heat conduction by hairs and tissues results in the following four typical *T*-levels of the skin surface (see the horizontal dotted lines in Fig. 6): *T*_i_ of skin surface below sunlit white hairs < *T*_ii_ of skin surface below sunlit white hairs and above a vessel < *T*_iii_ of skin surface below sunlit black hairs < *T*_iv_ of skin surface below sunlit black hairs and above a vessel. A small increase of *T* occurs above a vessel and the positive *T*-gradients point always toward the skin area directly above the vessel. Thus, on a skin surface with homogeneous-coloured hairs a vessel next to the skin surface can reliably be detected on the basis of these positive *T*-gradients (represented by green horseflies in Fig. 6A,B), independently of the pelage colour.

However, at the borderlines of sunlit black and white stripes of a striped pelage, similar *T*-gradients occur on a skin surface without an underlying vessel because of the hair-conducted warmth (Fig. 6C,D). These *T*-gradients can deceive vessel-seeking horseflies which thus bite the skin at the *T*-gradients and try to suck blood. Such a blood-sucking attempt is, of course, unsuccessful (represented by red horseflies in Fig. 6C,D), but elicits parasite-repelling reactions of the host animal, being very dangerous for the flies.

The above-mentioned unsuccessful blood-sucking attempts represented by red horseflies in Fig. 6 demonstrate well how and why the numerous borderlines of sunlit black-white stripes of zebras can make it more difficult to locate blood vessels, which are detected by thermoreceptors contacting skin beneath the hairs. These frequent false vessel locations, the subsequent painful bitings and the host’s fly-repellent reactions enhance considerably the chance that the fly cannot evade host responses and is swatted by them. To eliminate this risk, a good evolutionary strategy is the avoidance of striped (and spotted) hosts.

Our main assumption is that the gradients of temperature *T* at the borderlines of sunlit neighbouring black and white zebra stripes deceptively could mimick vessel-induced *T*-differences for blood-seeking female tabanids, if they look for vessels by thermoreception. A vessel induces a double *T*-gradient (i.e. increasing and then decreasing *T* when crossing the vessel, Fig. 6A,B) within the vessel’s diameter (ca. 1–5 mm), whereas the borderline of an adjacent sunlit black-white stripe pair induces a single *T*-gradient within 1–5 mm (i.e. increasing or decreasing *T* when crossing the borderline, Fig. 6C,D). Since the usual dimension of a horsefly is at least about 10 mm, a vessel-seeking female may be able to distinguish the double *T*-gradient from the single one. The question is whether the fly could discern a vessel on the basis of the vessel-specific double *T*-gradient.

### Tabanid-preference of black stripes on the sunlit half of the grey barrel

In our field experiment, host-seeking female tabanids landed on the black-striped grey barrel. Some of them walked for shorter or longer periods on the barrel’s surface. During walking they preferred the thin black stripes to the grey rectangles exclusively on the sunlit half of the barrel’s cylinder. This can be explained by the fact that the sunlit black stripes were slightly warmer than their sunlit grey surroundings, while the temperatures of the shady grey rectangles and the shady black stripes were practically the same. The walking horseflies could prefer the black stripes due to their darker appearance and/or larger degree of polarization and/or higher temperature relative to those of the grey areas. However, the stripe preference caused by the stripes’ different darkness and polarization is excluded by the finding that on the shady half of the barrel there was neither stripe preference, nor relevant temperature difference between black stripes and their grey surroundings.

The attractiveness of host animals to tabanids decreases with increasing inhomogeneity of the host’s coat pattern^32,40^. According to our hypothesis, one of the reasons for this is that the dark-bright borderlines are more numerous on a more inhomogeneous coat. The temperature *T* (dark: higher *T*, bright: lower *T*) of the sunlit body surface changes spatially suddenly perpendicular to these borderlines. Due to such a *T*-gradient at a sunlit borderline, a female horsefly searching for a blood vessel by means of thermal gradients can erroneously assume a vessel, because the dark area is warmer than the bright one, but there is actually no vessel there. On the skin surface above a vessel positioned next to the surface, a positive/negative *T*-gradient (i.e. increasing *T*) points toward/away from the vessel being slightly warmer than the surrounding tissues. Therefore, if a sunlit coat has a more inhomogeneous pattern, it is more difficult to find vessels with thermoreception, because the numerous *T*-gradients at the borderlines of stripes/spots have nothing to do with vessel-induced ones. Especially on the head and legs of zebras, the stripes are thin (< 1 cm), and these body regions should be defended the most against biting flies.

Under shady conditions, there are practically no temperature differences between shady dark and shady bright stripes/spots^39,42,44,57–59^, and therefore there are no temperature gradients at their borderlines. In this case, tabanids could find blood vessels by thermoreception on inhomogeneously coloured host animals as easily as on homogeneously coloured ones. However, blood-seeking female tabanids attack their hosts mainly in sunshine, because they prefer warmer hosts^42^, since they can escape easier from warmer targets^43^.

How do our results relate to previous studies showing that horseflies prefer warmer temperatures, because it helps them evade host responses? It is an observational and experimental fact that horseflies prefer sunlit homogeneous dark hosts against sunlit hosts with bright or heterogeneous (striped/spotted) coat patterns^6,32–35,40,41,47,54^. Tabanids avoid striped/spotted hosts, and usually do not land to seek for blood vessels on dark stripes/spots despite of their higher temperature in sunshine. This avoidance is an enigmatic behaviour, since on thicker dark/bright stripes or larger dark/bright spots—where only vessel-induced *T*-gradients occur on the skin surface below the hair layer—horseflies could find vessels by thermoreception as easily as on the skin of homogeneous dark/bright hosts. It is also an observational fact that the very few horseflies that land on sunlit striped/spotted targets alight mainly on the thick/large dark stripes/spots and avoid thin/small ones^32,40^. The reason for this may be twofold: they prefer thick/large dark sunlit stripes/spots because (i) due to the local higher temperature they can easier evade dangerous host responses, and (ii) they can easier find vessels by thermoreception due to the lack of *T*-gradients induced by the borderlines of stripes/spots. Thus, our results are consistent with the results of the mentioned previous investigations.

### Cue contrast between the black stripes and their grey surroundings on the sunlit and shady barrel

The intensity *I* of reflected light is proportional to the intensity *I*_0_ of incident light, and the proportionality factor is the reflectivity *R* (or albedo) of the surface at a given angle of incidence and wavelength λ: *I* = *R·I*_0_. Therefore, the intensity contrast *C*_int,grey_ = (*I*_grey_ – *I*_black_)/*I*_grey_ = (*R*_grey_ – *R*_black_)/*R*_grey_ or *C*_int,black_ = (*I*_grey_ – *I*_black_)/*I*_black_ = (*R*_grey_ – *R*_black_)/*R*_black_ between the black stripes and their grey surroundings is independent of the incident intensity *I*_0_ and depends only on the reflectivities/albedos *R*_grey_ and *R*_black_ of reflecting surfaces. Thus, if the incident intensity changes by a factor, because the sunlit black-striped grey barrel becomes shady or vice versa, then both intensities of light reflected from the black and grey surfaces also change by the same factor, and therefore the intensity contrast between the black stripes and the grey surroundings does not change. Consequently, the intensity contrast between the black stripes and their grey surroundings of our test barrel was practically the same for both the sunlit and the shady situations.

The polarized intensity *I*_pol_ of reflected light (i.e. intensity of the linearly polarized component of light) is proportional to the incident intensity *I*_0_. If *I*_0_ changes by a factor, because the shady black-striped grey barrel surface becomes sunlit, or vice versa, then the polarized intensities of light reflected from the black and grey surfaces also change by the same factor. Therefore, the degree of polarization *d* = *I*_pol_/*I*_0_ is independent of the incident intensity *I*_0_ and depends only on the polarizing capability of the reflecting surface at a given angle of incidence and wavelength λ. Since the degrees of polarization *d*_black_ and *d*_grey_ practically do not change when the sunny/shady barrel becomes shady/sunny, the polarization contrast *C*_pol,grey_ = (*d*_black_ – *d*_grey_)/*d*_grey_ or *C*_pol,black_ = (*d*_black_ – *d*_grey_)/*d*_black_ between the black stripes and their grey surroundings does not change. This is seen in the polarization patterns of our sunlit and shady barrel (Fig. 1, Supplementary Figs. S2, S3). Consequently, the polarization contrast between the black stripes and their grey surroundings of our test barrel was practically the same for both the sunlit and the shady barrel.

The degree of polarization *d* depends also on the angle of incidence β (e.g. measured from the normal vector of the surface). At both β = 0° and 90°, *d* = 0. If β increases from 0° to 90°, then *d* first increases, at the Brewster’s angle β_Brewster_ = arc tan (*n*) reaches its maximum *d*_max_, then decreases to 0, where *n* is the refractive index of the reflecting surface. Thus, for both the shady and the sunlit barrel, the maximum of the polarization constrast *C*_pol,grey_ = (*d*_black_ – *d*_grey_)/*d*_grey_ or *C*_pol,black_ = (*d*_black_ – *d*_grey_)/*d*_black_ between the black stripes and their grey surroundings can occur at β_Brewster_, while if β nears 0° or 90°, both *d*_black_ and *d*_grey_ nears zero, and therefore both polarization contrasts *C*_pol,grey_ and *C*_pol,black_ approximate zero, too. These polarization characteristics are also visible in the polarization patterns of our shady and sunlit barrels (Fig. 1, Supplementary Figs. S2, S3).

In the case of the shady barrel, skylight was the light source, while the sunlit barrel was predominantly illuminated by sunlight when skylight played only a marginal role. Depending on the sky’s cloudiness conditions and the elevation angle of the Sun above the horizon, the spectra of skylight and sunlight are more or less different^81–84^. Since our black-striped grey barrel has only colourless (black, grey) surface parts—the reflection spectra of which are approximately constant as the wavelength λ of light changes—, its reflection-polarization characteristics are practically independent of λ (Fig. 1, Supplementary Figs. S2, S3). Consequently, the intensity and polarization contrasts between the black stripes and their grey surroundings are practically the same for both the shady barrel (illuminated only by skylight) and the sunlit barrel (illuminated mainly by sunlight).

On the basis of the above optical arguments, we conclude that relevant difference between the sunlit and shady black-striped grey barrels was only in the surface temperature (the sunlit black stripes were warmer than their sunlit grey surroundings, while the intensity and polarization contrasts were practically the same on both the sunlit and the shady barrels), rather than also in the intensity and polarization. Consequently, our test target demonstrated only thermal effects, and the results can be explained unambiguously by this thermal difference.

## Supplementary Information

Supplementary Information.

## Data Availability

Our paper has the following Electronic Supplementary Material: Supplementary Figs. S1, S2, S3 and Supplementary Table S1.
