# Supplementary material for: Sunlit zebra stripes may confuse the thermal perception of blood vessels causing the visual unattractiveness of zebras to horseflies
Source: Sci Rep. 2022 Aug 4;12:10871. doi: 10.1038/s41598-022-14619-7 (PMC9352684; doi:10.1038/s41598-022-14619-7)
Supplement: Supplementary file 1 — Supplementary Information. [file 41598_2022_14619_MOESM1_ESM.docx]

**Electronic Supplementary Material**

for

**Sunlit zebra stripes may confuse the thermal perception of blood vessels causing the visual unattractiveness of zebras to horseflies**

Péter Takács, Dénes Száz, Miklós Vincze, Judit Slíz-Balogh, Gábor Horváth^1,^*

*Corresponding author: Department of Biological Physics, ELTE Eötvös Loránd University,

H-1117 Budapest, Pázmány sétány 1, Hungary, gharago.elte.hu

This file contains the following: Supplementary Table S1

Supplementary Figures S1, S2, S3

**Supplementary Table S1.** Sum and percentage (in brackets) of time period (second) spent by female tabanids walking on the grey areas and the black stripes of the sunlit (solar) and shady (antisolar) halves of the barrel’s mantle on each experimental day, furthermore the total and percentage of these times. s.d.: standard deviation.

| **sunlit (solar) half of the**  **barrel’s mantle** | | | **shady (antisolar) half of the**  **barrel’s mantle** | | |
| --- | --- | --- | --- | --- | --- |
| **experimental**  **day (2021)** | **grey areas** | **black stripes** | **experimental**  **day (2021)** | **grey areas** | **black stripes** |
| 10 July | 2417 s  (82.0 %) | 531 s  (18.0 %) | 7 July | 2563 s  (93.4 %) | 180 s  (6.6 %) |
| 18 July | 1916 s  (80.2 %) | 473 s  (19.8 %) | 21 July | 1519 s  (93.8 %) | 101 s  (6.2 %) |
| 22 July | 2071 s  (83.7 %) | 403 s  (16.3 %) | 23 July | 2309 s  (93.6 %) | 157 s  (6.4 %) |
| 24 July | 1465 s  (75.0 %) | 488 s  (25.0 %) | 25 July | 2647 s  (93.9 %) | 172 s  (6.1 %) |
| 26 July | 1349 s  (86.4 %) | 213 s  (13.6 %) | 27 July | 1644 s  (93.9 %) | 106 s  (6.1 %) |
| 28 July | 1493 s  (74.0 %) | 525 s  (26.0 %) | 29 July | 1677 s  (93.6 %) | 114 s  (6.4 %) |
| 31 July | 846 s  (83.0 %) | 173 s  (17.0 %) | 1 August | 1061 s  (93.8 %) | 70 s  (6.2 %) |
| 4 August | 748 s  (81.0 %) | 176 s  (19.0 %) | 3 August | 1396 s  (93.8 %) | 92 s  (6.2 %) |
| 5 August | 589 s  (77.1 %) | 175 s  (22.9 %) | 7 August | 1056 s  (91.7 %) | 95 s  (8.3 %) |
| 8 August | 497 s  (82.0 %) | 109 s  (18.0 %) | 9 August | 914 s  (93.7 %) | 61 s  (6.3 %) |
| **total** | **13391 s** | **3266 s** |  | **16786 s** | **1148 s** |
| **mean ± s. d.**  **of time ratios** | **80.4 ± 3.9 %** | **19.6 ± 3.9 %** |  | **93.6 ± 0.7 %** | **6.4 ± 0.7 %** |


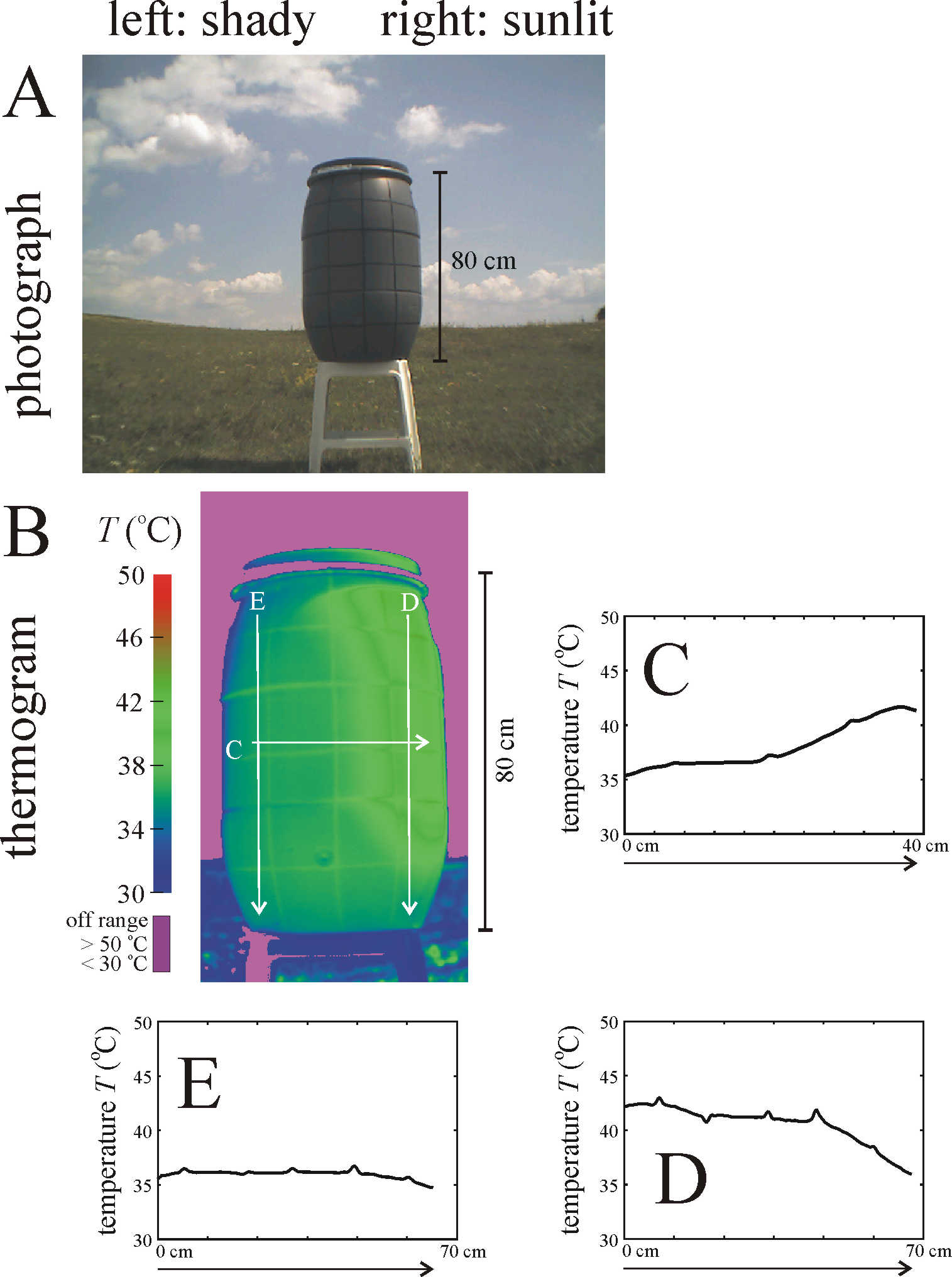


**Supplementary Figure S1.** Photograph (A) and thermogram (B) of the sunlit (right half) and shady (left half) black-striped grey barrel used in experiment 1 and illuminated by direct sunlight from the right hand side. The barrel’s surface temperature *T* (^o^C) along the horizontal and vertical arrows in the thermogram are shown in panels C, D and E.


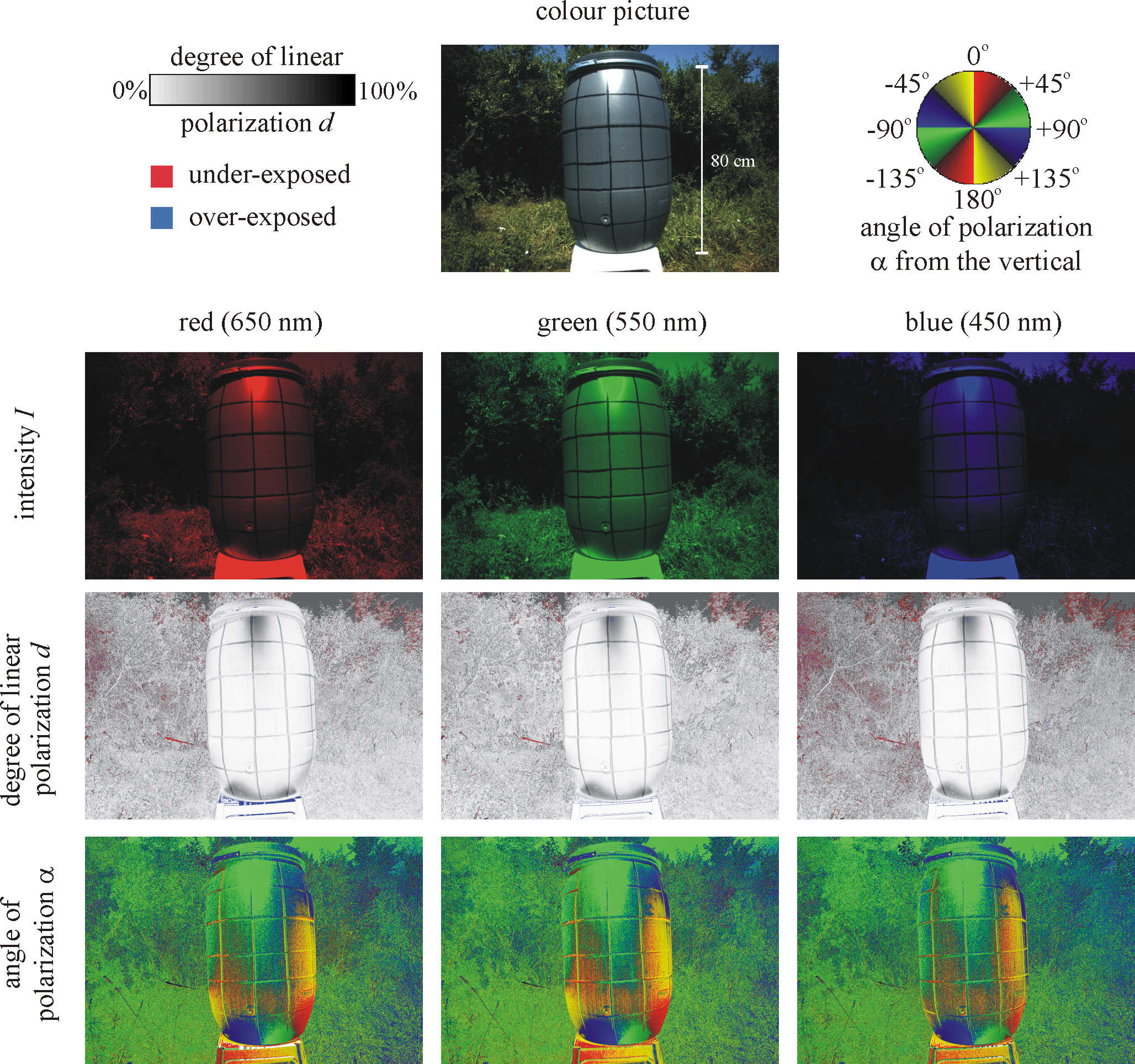


**Supplementary Figure S2.** Reflection-polarization patterns of the sunlit black-striped grey barrel used in field experiment 1 measured in the red (650 ± 40 nm), green (550 ± 40 nm) and blue (450 ± 40 nm) parts of the spectrum when the optical axis of the polarimeter was horizontal.


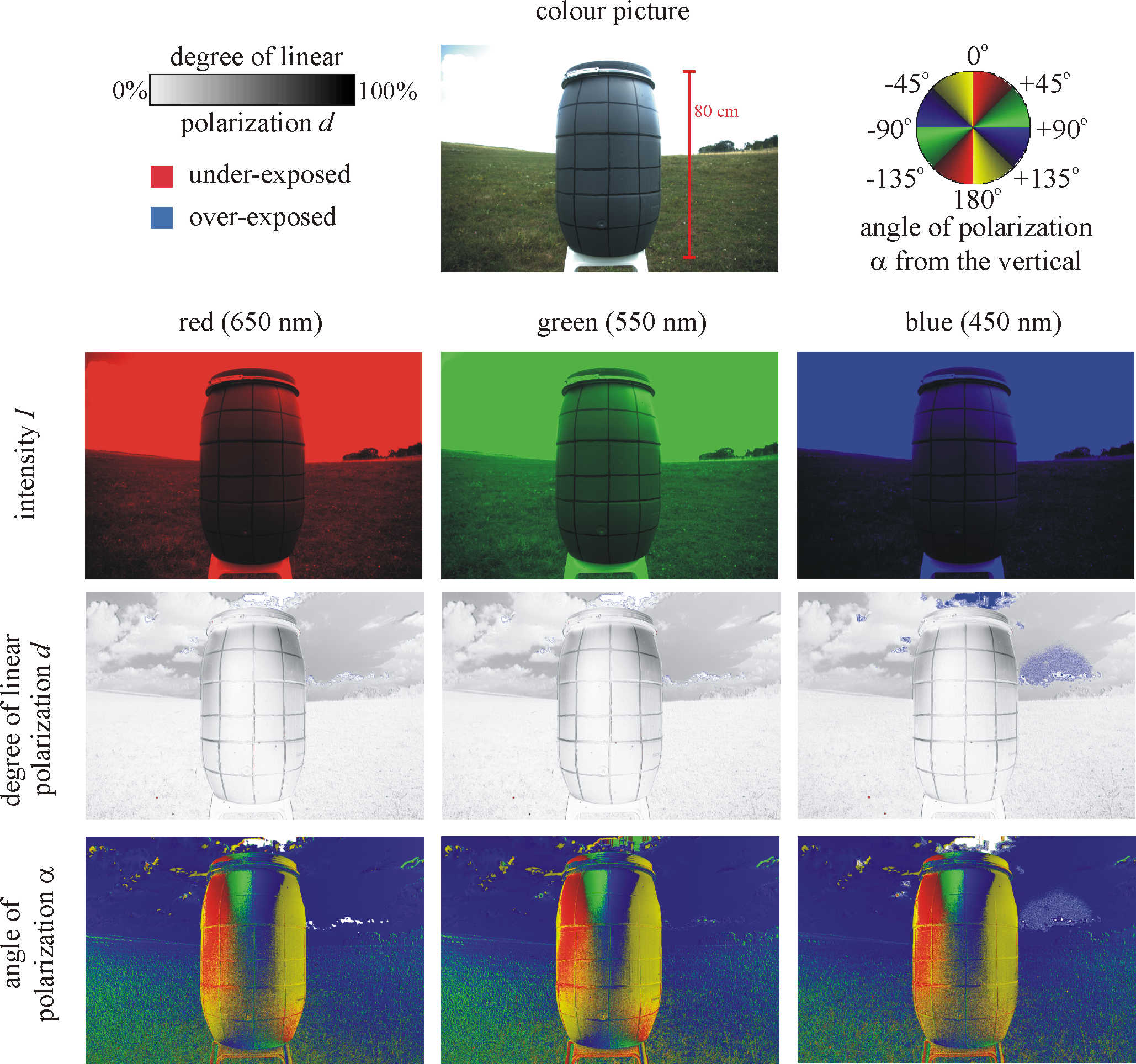


**Supplementary Figure S3.** Reflection-polarization patterns of the shady black-striped grey barrel used in field experiment 1 measured in the red (650 ± 40 nm), green (550 ± 40 nm) and blue (450 ± 40 nm) parts of the spectrum when the optical axis of the polarimeter was horizontal.
